# Case Report: A case of pediatric persistent refractory ITP responsive to avatrombopag

**DOI:** 10.3389/fimmu.2025.1576053

**Published:** 2025-04-15

**Authors:** Antonella Sau, Gianfranco Di Prinzio, Daniela Onofrillo, Anna Caterina Russo, Nicole Santoro, Mauro Di Ianni

**Affiliations:** ^1^ Department of Hematology and Oncology, Santo Spirito Hospital, Pescara, Italy; ^2^ Department of Medicine and Sciences of Aging, G. d’Annunzio University of Chieti and Pescara, Chieti, Italy

**Keywords:** immune thrombocytopaenia (ITP), avatrombopag, thrombopoietin receptor (TPOr) agonists, children, platelet, persistent or chronic immune thrombocytopenia

## Abstract

Immune thrombocytopenia (ITP) is a rare hematologic disorder characterized by low platelet counts due to an immune-mediated destruction of platelets. While corticosteroids, intravenous immunoglobulin (IVIG) are the mainstays of treatment, a subset of patients may remain refractory to these therapies. Here, we present a case of a 6-year-old girl diagnosed with refractory ITP, who failed to respond to standard therapies but showed a remarkable clinical improvement with avatrombopag, a thrombopoietin receptor agonist.

## Introduction

Pediatric primary immune thrombocytopenia (ITP) is an acquired immune-mediated bleeding disorder characterized by an isolated platelet count decrease without an identified cause during childhood (1). The incidence of pediatric ITP is estimated to be 1.6–5.3 per 100,000 children annually, with a higher prevalence observed in younger age groups (2, 3).

Despite extensive research, the precise etiology of ITP remains elusive, with genetic, environmental, and immunological factors all contributing to its development (4). Clinically, children with ITP present with symptoms ranging from mild bruising and petechiae to severe hemorrhagic events, significantly affecting their quality of life (2).

Current management strategies for pediatric ITP focus on balancing the risks of bleeding with the side effects of treatment. Although many children may have a spontaneous remission or respond to first-line treatments such as corticosteroids and IVIG, refractory cases may require second-line treatments. Currently, second-line therapy has shifted from immunosuppressive agents to thrombopoietin receptor agonists (TPO-RAs) (5, 6).

Thrombopoietin (TPO) is a hormone constitutively produced by the liver, which regulates platelet production by binding to and activating TPO receptors on the megakaryocyte cell surface, thereby inducing intracellular signalling cascades that lead to increased platelet production (7). Plasma levels of TPO are regulated by binding of TPO to circulating platelets, which results in its removal from circulation and subsequent degradation. In patients with ITP, TPO plasma levels are inappropriately low as compared with individuals with hypoproliferative thrombocytopenias, an observation that led to the development of recombinant thrombopoietins, the first generation of exogenous thrombopoiesis-stimulating agents (8).

Avatrombopag is a small new molecule TPO-RA that mimics the biological effects of TPO *in vitro* and *in vivo* (9). Because of its unchelated polyvalent cations, it can be administered orally with food, does not cause hepatotoxicity and improves compliance and convenience in children with ITP (10).

This case highlights the effectiveness of avatrombopag in a child who was refractory to all conventional treatments.

## Case presentation

This 6-year old girl presented in April 2024 with widespread petechiae and a platelet count of 1.000/mm³. She underwent initial treatment with IVIG (1g/Kg for 1 day), with no improvement. Bone marrow aspiration and biopsy suggested no evidence of malignancy or marrow failure, therefore a short-term therapy with prednisone was prescribed (1 mg/Kg/daily for 14 days). Furthermore, autoimmunity test and autoimmune lymphoproliferative syndrome (ALPS) panel have been excluded. The patient displayed persistent severe thrombocytopenia and hemorrhagic manifestations despite treatment. Given the refractoriness, a second line treatment with first generation TPO-RA (eltrombopag 75 mg/die) was initiated.

On 16th May 2024 the patient was still thrombocytopenic and we decided to start a combination therapy with TPO-RA and immunosuppressive agent (mycophenolate-mofetil) but the patient failed to achieve response (even just a slight increase in plt).

Between May and October 2024, the patient was hospitalized 3 times for severe thrombocytopenia with mucocutaneous bleeding and performed on demand steroid therapy with Dexamethasone (12 mg daily) for 4 consecutive days, which led to a transient increase in platelet count.

Due to a decrease in platelet count, the patient started romiplostim 1 microg/Kg sc in August 2024 with a progressive escalation of dose until 10 μg/kg. Since the patient failed to achieve a response, romiplostim was discontinued at the end of November 2024.

At that time the patient quality life was compromised by frequent hospital admissions, absence from school and concern about recurrent bleeding; Furthermore the 120 Km distance from the reference center made the situation even more complicated.

**Figure 1:**
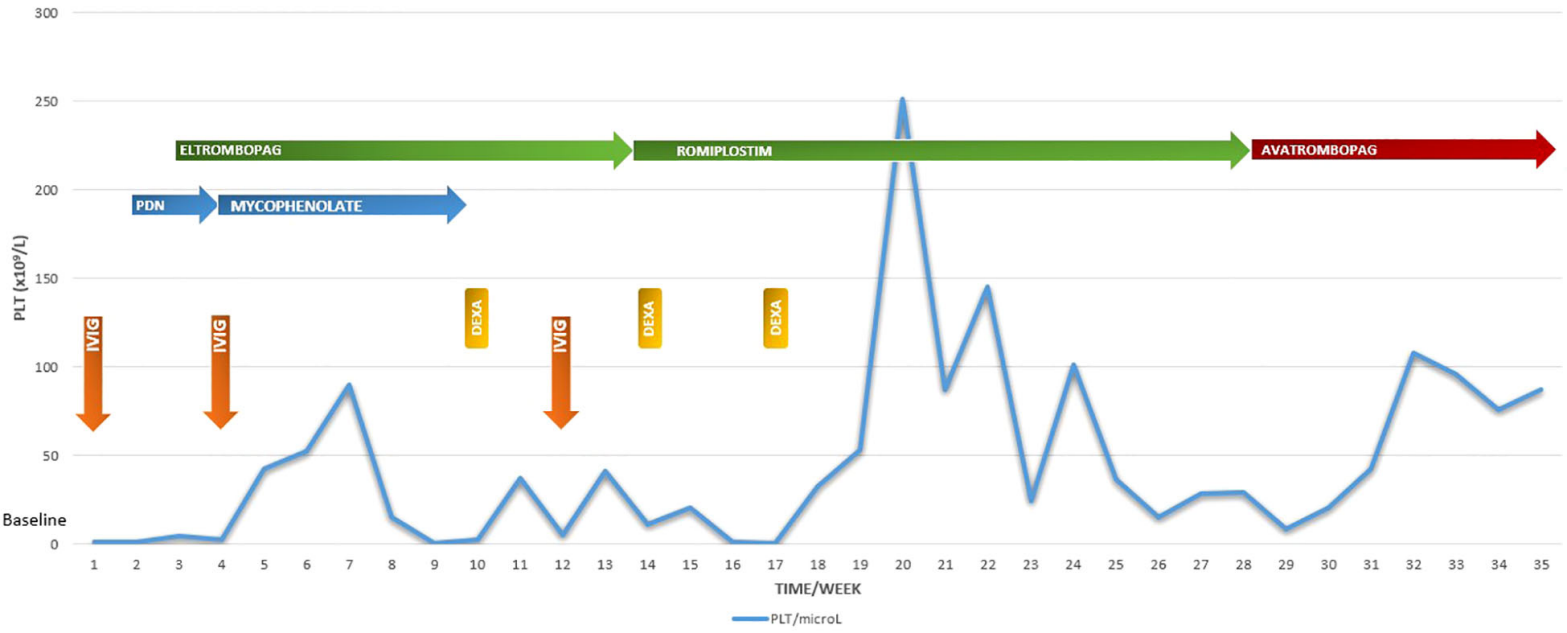
Platelet counts at every scheduled visit during the 8-month follow-up period in relation to the ongoing treatment. PLT platelet: IVIG IntraVenous ImmunoGlobulin: DEXA dexamethasone: PDN prednisone.

At December 2024, we decided to try a new alternative therapy and, after approval of an off-label use by ethics committee, avatrombopag was initiated at a starting dose of 20 mg/day; the drug dose was adjusted based on the clinical response and the platelet monitoring (20 mg three times a week). Platelet count began to rise steadily, reaching 50,000/mm³ within two weeks (**Figure 1**).

In January 2025 the platelet count stabilized above 100,000/mm³ with no additional bleeding episodes.

## Discussion

This case highlights the complex management of refractory ITP (r-ITP) particularly in pediatric patients. r-ITP is defined as the absence of no response to > 2 first –line treatments, it may evolve or not toward chronic immune thrombocytopenia or refractory chronic immune thrombocytopenia (11).

This patient refractoriness to standard treatments, including corticosteroids, IVIG, and TPO-RAs like eltrombopag and romiplostim, highlights the need for alternative therapies.

Treatment choice in children with refractory ITP can be challenging. As an alternative to TPO-RAs, rituximab and splenectomy can be evaluated but both treatment options may have significant adverse events.

Specifically, rituximab, despite an overall reassuring safety profile, may have several potential risks to be considered, including treating patients with underlying immunodeficiency, as well as toxicities such as ipogammaglobulinemia and neutropenia.

Recently FDA and EMA approved avatrombopag, an oral small-molecule TPO-RA, for the treatment of primary chronic ITP in adult patients. Its oral administration with or without food and the predictable pharmacokinetics make it particularly suitable for pediatric use. At the moment it is not approved in the pediatric population. Although, a multicenter retrospective observational study was conducted in children with persistent or chronic ITP that demonstrated the efficacy and safety of avatrombopag in patients who have failed or relapsed on previous treatment (1).

## Conclusion

Avatrombopag proved to be an effective and well-tolerated option for this pediatric patient with refractory ITP, achieving sustained platelet counts and preventing further bleeding episodes. This case supports its potential utility in similar clinical scenarios.

The usage of this drug in pediatric setting offers the advantage of an easy-to-use and effective therapy that can be administered at home, reducing the inconveniences associated with numerous venous sampling and hospital admissions; it can represent a valuable alternative to more aggressive therapies such as rituximab and splenectomy, that are difficult to propose in the early stages of the disease.

Conflict of Interest Statement the authors declare no conflicts of interest related to this case report.

## Data Availability

The original contributions presented in the study are included in the article/supplementary material. Further inquiries can be directed to the corresponding author.
